# Paternal Cyclophosphamide Exposure Induces the Formation of Functional Micronuclei during the First Zygotic Division

**DOI:** 10.1371/journal.pone.0027600

**Published:** 2011-11-16

**Authors:** Lisanne Grenier, Bernard Robaire, Barbara F. Hales

**Affiliations:** 1 Department of Pharmacology and Therapeutics, McGill University, Montréal, Canada; 2 Department of Obstetrics and Gynecology, McGill University, Montréal, Canada; Faculty of Pharmacy, Ain Shams University, Egypt

## Abstract

Paternal exposures to cancer chemotherapeutics or environmental chemicals may have adverse effects on progeny outcome that are manifested in the preimplantation embryo. The objectives of this study were to determine the impact of paternal exposure to cyclophosphamide, an anticancer alkylating agent, on the formation, chromatin origin and function of micronuclei in cleavage stage rat embryos. Male Sprague-Dawley rats were gavaged with saline or cyclophosphamide (6 mg/kg/day) for 4 weeks and mated to naturally cycling females to collect pronuclear zygotes and 2 to 8 cell embryos. Micronuclear chromatin structure was characterized using confocal microscopy to detect immunoreactivities for H3K9me3, a marker for maternal chromatin, and lamin B, a nuclear membrane marker. DNA synthesis was monitored using EdU (5-ethynyl-2′-deoxyuridine) incorporation. Fertilization by cyclophosphamide-exposed spermatozoa led to a dramatic elevation in micronuclei in cleavage stage embryos (control embryos: 1% to 5%; embryos sired by treated males: 70%). The formation of micronuclei occurred during the first zygotic division and was associated with a subsequent developmental delay. The absence of H3K9me3 indicated that these micronuclei were of paternal origin. The micronuclei had incomplete peri-nuclear and peri-nucleolar lamin B1 membrane formation but incorporated EdU into DNA to the same extent as the main nucleus. The formation of micronuclei in response to the presence of a damaged paternal genome may play a role in increasing the rate of embryo loss that is associated with the use of assisted reproductive technologies, parenthood among cancer survivors, and paternal aging.

## Introduction

The incidence of cancer in man of reproductive age has been on the rise over the past few decades; consequently, treatment with chemotherapeutics and radiotherapy is now common amongst the younger male population [1]. Many of these young men survive and experience compromised fertility; their decrease in the likelihood of fathering a child is dependent on the treatment regimen [2]–[4]. Cyclophosphamide (CPA) is a commonly used chemotherapeutic agent that is an alkylating agent and induces DNA double strand breaks [5]. Previous studies, from our laboratories and others, have shown that the treatment of male rats with CPA has dose dependent and time specific effects on spermatogenesis with adverse effects on embryo development [6]–[9]. The greatest amount of DNA damage in male germ cells exposed to CPA was observed when elongating spermatids were targeted; when CPA-exposed male rats were mated to untreated, healthy females, a marked delay in the formation of blastocysts was observed, followed by an increase in peri-implantation embryonic loss [6], [10]. Indeed, a DNA damage response was activated in the early post-fertilization zygote [11].

Exposure of various cell types to radiation or to a wide range of chemicals damages DNA or disrupts microtubules and spindle assembly, resulting in chromosomal aberrations and the formation of micronuclei [12], [13]. Micronuclei are small, extranuclear, DNA containing structures. In toxicology, the formation of micronuclei is used as an in vitro assay to detect putative chemical mutagens or carcinogens [14]. However, recent studies have revealed that non-genotoxic chemicals, such as retinoic acid, may also induce the formation of micronuclei in pluripotent stem cells, suggesting an association between the formation of micronuclei and neural differentiation [15]. Thus, the formation and fate of micronuclei may have important implications for both the genomic stability and plasticity of cells.

Studies with cancer patients and using animal models have revealed that exposure to cancer chemotherapeutics during germ cell formation in the testis or maturation in the epididymis may adversely affect the quality of spermatozoa, as assessed by a variety of sperm quality tests [16], [17]. Spermatozoa with damaged chromatin are capable of fertilizing oocytes, thus transferring this lesion to the zygote [18]. Indeed, paternal exposure to acrylamide, a chemical found in tobacco smoke and produced during the cooking of starchy foods, increased the formation of micronuclei in two cell embryos, during the first mitotic division, resulting in chromosomal mosaicism [19]. Intracytoplasmic sperm injection was associated with abnormal chromosome segregation and micronuclear formation in two-cell stage mouse embryos [20]. Despite the extensive use of assisted reproductive techniques, the numbers of live offspring produced after intracytoplasmic sperm injection are low [20], [21]. Thus, it is important to investigate the possible consequences of micronuclear formation on events in early embryos. The goal of this study was to determine the impact of paternal exposure to cyclophosphamide on early cleavage stage embryo development and on the formation, chromatin origin and function of micronuclei.

## Materials and Methods

### Drug treatment and embryo collection *in vivo*

#### Ethics Statement

This study was done in accordance with the guidelines of the Canadian Council on Animal Care for the ethical use and care of animals in science. The protocol (Protocol Number: 2144) was approved by the Animal Care Committee of McGill University.

Adult male (350–400 g) and virgin female (225–250 g) Sprague-Dawley rats were purchased from Charles River Canada (St. Constant, Quebec, Canada) and housed at the Animal Resources Centre, McIntyre Medical Building, McGill University (Montreal, Canada). Animals received food and water *ad libitum* and were maintained on a 0700–1900hr light/dark cycle. The drug treatment and zygote protocols previously described [6] were followed with minor modifications. After one week of acclimatization, male rats were randomly assigned to one of two treatment groups and gavaged with saline (vehicle) or CPA (CAS 6055-19-2; Sigma Chemical Co., St Louis, Missouri), 6 mg/kg per day, six times per week for four weeks.

On the fifth week of treatment, proestrus control virgin females were selected by a vaginal wash in the mid-afternoon and were caged overnight, in groups of two, with either a control or CPA treated male. Pregnancies were confirmed with sperm positive vaginal smears the following morning, designated as gestation day 0. Sperm positive females were euthanized on day 0 at 1300hr, on day 1 at 1000hr, on day 2 at 1400hr and day 3 at 1000hr to collect pronuclear zygotes, 2 cell, 4 cell and 8 cell embryos, respectively. Oviducts and whole uteri were isolated and cleaned in pre-warmed (37°C) M2 culture medium (Sigma Chemical Co.), pronuclear zygotes were collected from the ampullae in warm M2 medium, and early cell cleavage embryos were flushed with a 30 round gauge needle from the infundibulum of the oviduct with 0.2 ml of warm M2 medium.

### Immunofluorescence

The immunofluorescence protocols used were described previously [22]. To characterize micronuclei from embryos sired by CPA exposed males, embryos were incubated with rabbit polyclonal anti-lamin B1 (1∶500 dilution; catalogue number ab16048, Abcam, Cambridge, MA) or rabbit polyclonal anti-trimethyl-histone H3 (Lys9) (1∶200 dilution; catalogue number 07-442, Millipore, Billerica, MA) overnight at 4°C in a humidified chamber. Both primary and secondary antibodies were diluted in goat blocking solution (10% goat serum, 3% BSA and 0.1% Tween 20 in PBS). Zygotes were then washed 3×15 min in goat blocking solution, incubated for 1 hr at room temperature with the secondary antibody, goat fluorescein anti-rabbit IgG (H+L) (1∶200 dilution; catalogue number F1-1000, Vector Laboratories, Burlington, Ontario, Canada), and rewashed 3×15 min in goat blocking solution. DNA was stained with propidium iodide (catalogue number P4864, Sigma Chemical Co.) at 10 µg/mL in goat blocking solution for 20 min, washed in 0.05% Tween 20 in PBS for 10 min, mounted in 3 µl of VectaShield mounting medium (Vector Laboratories) on a premarked pap pen slide and covered with a cover slip. Slides were then stored at 4°C and visualized with confocal microscopy within two days.

### In vitro zygotic development and detection of DNA synthesis

In vitro zygotic development was followed using the mR1ECM milieu [23]. To detect DNA synthesis, the Click-It kit (catalogue number C10337, Invitrogen, Burlington, Ontario, Canada) was used. A final concentration of 100 µM EdU (5-ethynyl-2′-deoxyuridine) was supplemented to mR1ECM+BSA (pH equilibrated to 7.4 with 10N HCl). This zygotic development milieu was covered with light mineral oil to prevent evaporation and pre-equilibrated for 3 h before its use in an incubator at 37°C and 5% CO_2_. All solutions were prepared fresh the day of the experiment.

### Embryo collection for in vitro incubation

Sperm positive females were euthanized at 1100hr on day 0 to collect pre-pronuclear stage zygotes in prewarmed (37°) M2 culture medium (Sigma Chemical Co.). Zygotes were released from the ampullae or flushed into a drop of prewarmed (37°C) 1% hyaluronidase (Sigma Chemical Co.) in M2 medium to digest the cumulus cells, washed three times in prewarmed (37°C) mR1ECM+BSA, and incubated in pre-equilibrated (37°C and 5%CO2) mR1ECM+BSA+100 uM EdU. The embryos were incubated overnight and analyzed under the light microscope every 15 min between 0900 and 1030hr on day 1 to assess the proportion of zygotes that divided to the 2 cell stage and thus completed the first zygotic division. To assess DNA synthesis in embryos collected on day 3 at 1000hr embryos were incubated in vitro for three hours. All of these steps. from the time of embryo collection to the in vitro incubation, were done within 7 min to preserve embryonic quality. Following collection of the embryos, EdU incorporation was detected as described below.

### Click-iT EdU Immunodetection

For a more complete Click-iT protocol please refer to the instruction manual from Invitrogen (catalogue number C10337). Embryos were washed in 3% BSA in 1× PBS (pH 7.4; Mg^2+^ and Ca^2+^ free). Zona pellucidae were removed in prewarmed (37°C) acid Tyrode solution (pH 2.5) by pipetting up and down for 5 sec, and then washed in 3% BSA in 1× PBS (pH 7.4; Mg^2+^ and Ca^2+^ free). During the permeabilization step, the Click-iT reaction cocktail was prepared in the following sequence: 1×Click-iT reaction buffer, 430 µl; CuSO4, 20 µl; Alexa Fluor azide, 1.2 µl; reaction buffer additive, 50 µl, and used within 15 min of preparation. Following the permeabilization step, the embryos were washed in 3% BSA in 1× PBS (pH 7.4; Mg^2+^ and Ca^2+^ free) and incubated for 30 min at room temperature with the Click-iT reaction cocktail. DNA was stained with propidium iodide (catalogue no. P4864, Sigma Chemical Co.) at 10 µg/ml in 3% BSA in 1× PBS (pH 7.4; Mg^2+^ and Ca^2+^ free) for 20 min and washed 1× PBS (pH 7.4; Mg^2+^ and Ca^2+^ free) twice, mounted in 3 µl of VectaShield mounting medium (Vector Laboratories) on a premarked pap pen slide, and covered with a cover slip. Slides were stored at 4°C and visualized by confocal microscopy during the next two days.

### Confocal microscopy

A Zeiss LSM 510 Axiovert 100 M confocal microscope with a Plan-Apochromat ×63/1.4 oil DIC objective was used to visualize the fluorescence of early post-fertilization zygotes. The best settings for laser scanning fluorescence imaging were determined experimentally for all primary antibodies and maintained for all cell cleavage stage embryos. All zygotes were scanned at a speed of 5–7 with an optical slice of 0.6 µm, zoom factor equal to one and a pinhole setting of 96 µm. Two scans of each optical section were compiled and averaged by the Zeiss LSM 510 computer software to give a final image that was 1024×1024 pixels in size. The embryonic cell cleavage stage was determined by counting the number of nuclei stained with propidium iodide and confirmed with phase contrast images. Qualitative analysis of embryo images was done for histone H3 trimethylated at K9 (H3K9me3) immunoreactivity to determine the parental origin of the chromatin (staining positive for maternal/female chromatin and negative for paternal/male chromatin) [24]. Analysis of the embryo images for lamin B1 reactivity, to characterize the nuclear and micronuclear membranes [25], [26], was also qualitative. The EdU embryo images from Z-stacks were further analyzed and quantified using the Imaris image analysis program version 7.2.3.

### Quantitative analysis

Polyspermic pronuclear zygotes were excluded from this analysis. To assess in vitro development, we counted the number of 2 cell embryos and divided this by the total number of fertilized oocytes, determined by propidium iodide nuclear staining, per replicate every 15 min between 0900 and 1030hr on day 1 (in vitro progression of the first zygotic division, SAL N = 5 to 8 males with total of 76 to 97 embryos assessed; CPA N = 2 to 5 males, with total of 31–75 embryos). To assess in vivo progression through early cell cleavage stages, based on propidium iodide nuclear staining, embryos were subdivided into four groups: 2, 3–4, 5–8 and 9–16 cells (on day 1 we collected SAL N = 8, 115 embryos, CPA N = 5, 56 embryos, day 2 SAL N = 4, 40 embryos, CPA N = 3, 26 embryos, day 3 SAL N = 8, 135 embryos, CPA N = 8, 120 embryos). The same embryos were analyzed for the incidence and average number of micronuclei per embryo and per cell. Micronuclei were differentiated from the main nucleus based on their smaller volume, with a similar nuclear morphology. Quantitative analysis of the nuclear and micronuclear volumes was done on day 0.5, with SAL N = 6, 109 embryos, CPA N = 5, 84 embryos; day 1, with SAL N = 8, 115 embryos, CPA N = 5, 64 embryos; and day 3, with SAL N = 8, 129 embryos, CPA N = 8, 120. Qualitative characterization of micronuclei with H3K9me3 and lamin B1 staining was done with SAL and CPA N = 2–3 with 15–30 embryos per group per embryonic cell cleavage stage. Quantification of in vitro overnight incubation with EdU for immunodetection on day 0–1 was done with SAL N = 6, 93 2 cell embryos CPA N = 5, 29 2 cell embryos; on day 3, the analysis of EdU after a 3 h in vitro incubation with EdU in 8 cell embryos was done with SAL N = 5, 55 embryos CPA N = 6, 73 embryos.

The Imaris image analysis program (Bitplane Inc., South Windsor, CT) was used to quantify the data on EdU immunodetection and the nuclear and micronuclear volumes. Basically, a nuclear and micronuclear surface was created from propidium iodide staining for each embryo to measure the average volume and average DNA content and a second nuclear and micronuclear surface was created from EdU staining to measure the average EdU incorporation per embryo. The data were graphed as the intensity mean per embryo per replicate.

### Statistical analyses

Chi squared analysis and the Fisher exact test, with Bonferroni correction when needed, were done to compare the proportion of embryos progressing either in vitro or in vivo between and within treatment groups, for the incidence of micronuclei and the proportion of cells that were EdU positive or contained EdU positive micronuclei during specific cell cleavage stages. Kruskal-Wallis analysis, with Bonferroni correction when needed, was done to determine the numbers of micronuclei per embryo and per embryonic cell, the transient volume changes and the micronuclear to nuclear volume ratios; these methods were also used to analyze EdU nuclear intensity means, DNA intensity means and their micronuclear to nuclear intensity ratios in early cell cleavage stage embryos between and within treatment groups. All statistical analyses were done using Systat (program version 10.2). Values are reported either as an average proportion or number per embryo per replicate ± standard error of the mean.

## Results

### Paternal exposure to cyclophosphamide affects the in vitro timing of the first zygotic division

**Figure 1:**
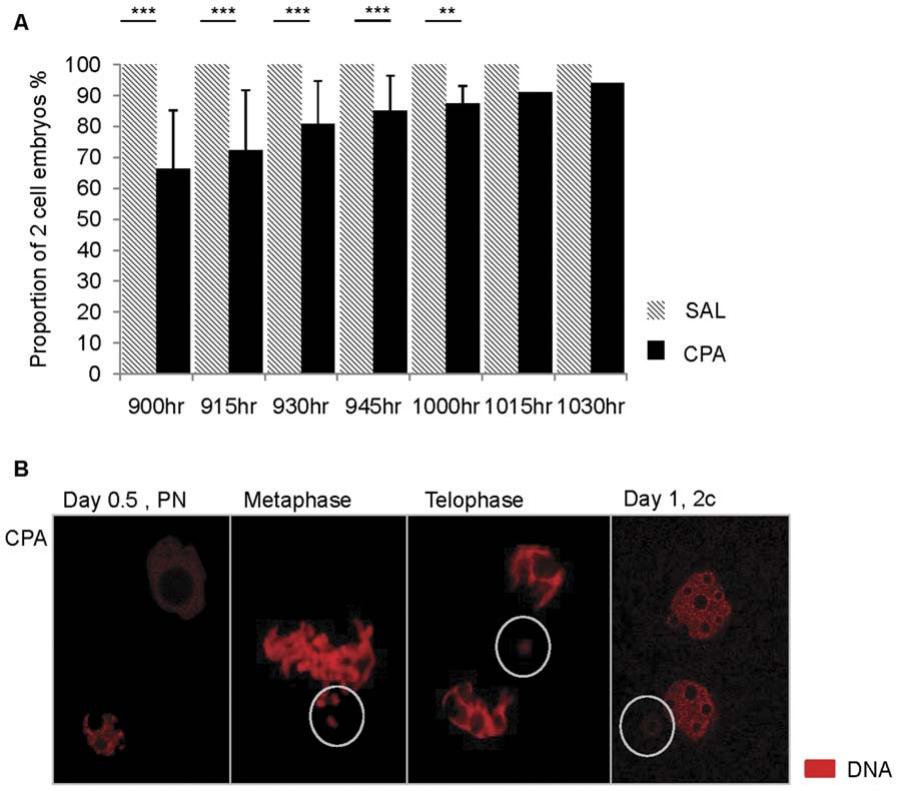
In vitro timing of the first zygotic division. (A) The proportion of fertilized oocytes that reached the 2 cell stage as assessed by light microscopy at 15 min intervals between 0900 and 1030hr on day 1. Paternal exposure to CPA delayed the timing of the first zygotic division without affecting their capacity to divide. Bar graphs represent the means per replicate ± standard errors of the mean; controls are in hatched bars and CPA-sired embryos in black bars. (B) Immunofluorescence images of CPA-sired embryos cultured in vitro and stained with propidium iodide, in red. Lagging pieces of chromatin are visible during the first metaphase and telophase of the zygotic cell cycle and micronuclei (MN) are clearly visible in 2 cell embryos. MN are formed during the first zygotic division in zygotes fertilized by CPA-exposed spermatozoa. Lagging chromatin and MN are circled in white. We collected SAL N = 5–8 males, 76 to 97 embryos and CPA N = 2–5 males, 31–75 embryos. Data were statistically analyzed by Chi-square, Fisher Exact test with Bonferroni's correction ** *P*≤0.01, *** *P*≤0.001.

The overnight in vitro incubation of pre-pronuclear zygotes allowed us to follow the timing of the first zygotic division. Paternal exposure to CPA significantly delayed the appearance of 2 cell embryos between 0900 and 1000hr on day 1 without affecting the capacity of all embryos to divide to the 2 cell stage by 1015hr (Fig. 1*A*). On day 1 at 0900hr, 100% of control versus 65% of CPA sired embryos had divided to the 2 cell stage; the remaining CPA sired embryos (35%) were actively undergoing mitosis, gradually reaching the 2 cell stage (Fig. 1*A*).

Lagging, fragmented pieces of condensed chromatin were clearly visible at metaphase and telophase during the first zygotic division of in vitro incubated CPA sired embryos; in contrast, pronuclear zygotes did not show any sign of fragmented pieces of DNA (Fig. 1*B*). It is highly likely that micronuclei are formed from this fragmented chromatin (Fig. 1*B*, 2 cell embryo). The morphology of the micronuclei in interphase stage 2 cell embryos was very similar to that of the nuclei; micronuclei were round with a nucleolus, had a smaller volume and a lower DNA staining intensity, compared to the main nucleus (Fig. 1*B*).

### Effects of paternal exposure to cyclophosphamide on the in vivo progression of early cell cleavage stage embryos

**Figure 2:**
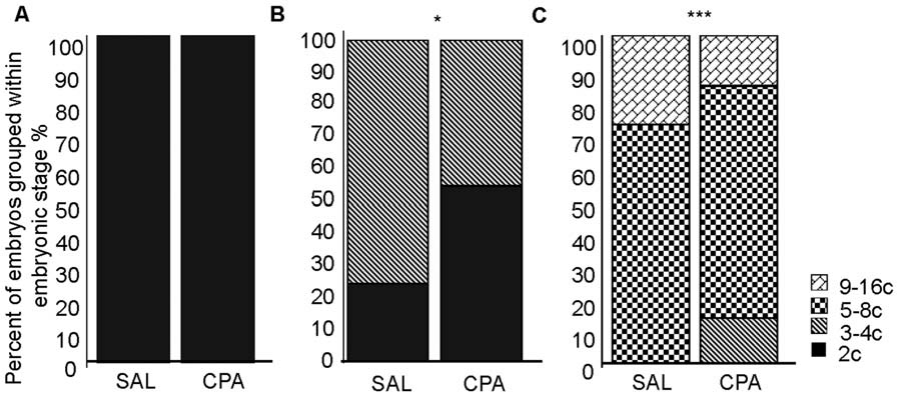
In vivo progression of early cell cleavage stage embryos. Early cell cleavage embryos were collected following in vivo fertilization on (A) day 1 at 1000hr; (B) day 2 at 1400hr; (C) day 3 at 1000hr. Embryonic stages were determined by counting the number of nuclei and cells using confocal microscopy, grouped within developmental stages (refer to the figure legend) and are reported as an average percent per replicate; error bars represent the standard errors of the mean. Paternal exposure to CPA delayed the progression of early cleavage stage embryos on day 2 and day 3 of collection. We collected: (A) day 1, SAL N = 8 males, 115 2-cell embryos, CPA N = 5 males, 56 2-cell embryos; (B) day 2, SAL N = 4 males, 43 embryos, CPA N = 3 males, 36 embryos; (C) day 3, SAL N = 8 males, 135 embryos, CPA N = 8 males, 120 embryos. Data were statistically analyzed with Chi-square, Fisher exact test for statistical analysis * *P*≤0.05, *** *P*≤0.001.

The developmental progression of cell cleavage embryos was significantly delayed in embryos fertilized by CPA exposed males (Fig. 2). Embryos collected on day 1 were all at the 2 cell stage (Fig. 2*A*). In contrast, on collection day 2, 23% of control embryos, compared to 55% of CPA sired embryos, were still at the 2 cell stage (Fig. 2*B*, *P*≤0.05)). On day 3 of collection, the delay in the progression of early cell cleavage embryos was even more significant (*P*≤0.001); none of the control embryos were at the 3–4 cell stage as opposed to 14% of the CPA sired embryos; 71% of both control and CPA sired embryos were at the 5–8 cell stage, while 29% of control compared to only 15% of the CPA sired embryos had progressed to form 9–16 cell stage embryos (Fig. 2*C*).

### The incidence of micronuclei in control and CPA sired cell cleavage embryos

**Figure 3:**
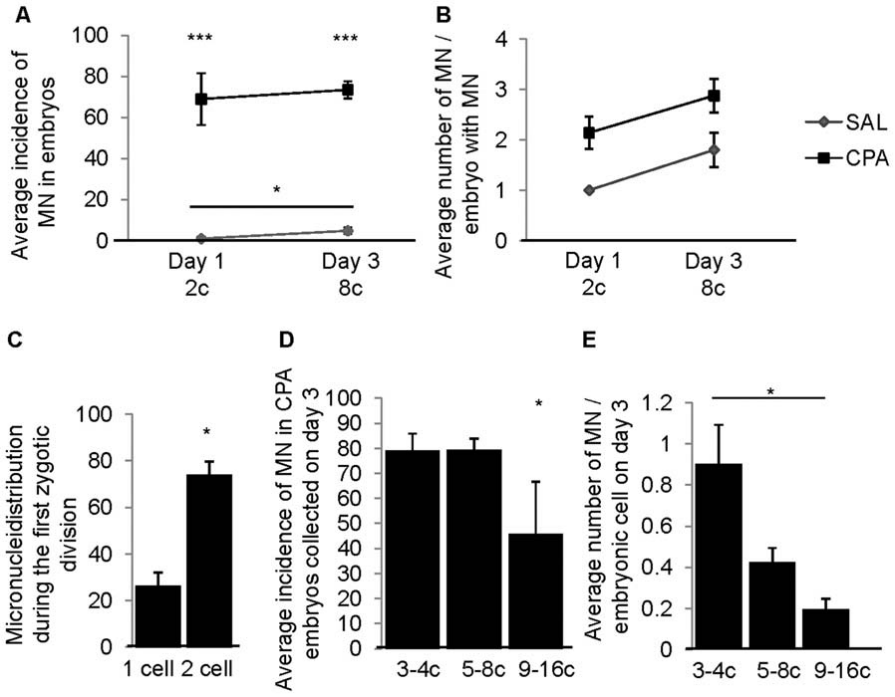
The incidence of micronuclei in cell cleavage embryos. (A) The incidence of micronuclei (MN) in cell cleavage embryos. (B) The average number of MN per embryo with MN. Paternal exposure to CPA significantly increased the incidence of MN in early cell cleavage embryos with an average of 2–3 MN per embryo compared to control. C) MN distribution following the first zygotic division in 2 cell embryos containing at least 2 MN. MN induced by paternal exposure to CPA were distributed randomly into the two cells during the first zygotic division. (D) The incidence of MN and (E) the average number of MN per embryonic cell in day 3 embryos sired by CPA treated males. The incidence of MN and number of MN per cell were significantly greater in delayed 3–4c embryos compared to normally dividing 5–8c and rapidly dividing 9–16c embryos. Line graphs are the average per replicate and error bars are standard errors of the means; the gray line represents SAL and black line CPA-sired embryos. We collected: day 1 SAL N = 8 males, 115 2 cell embryos, CPA N = 5 males, 56 2 cell embryos; day 3 SAL N = 8 males, 135 embryos, CPA N = 8 males, 120 embryos. The incidence of MN and average number of MN were analyzed by Chi-square Fisher Exact test and Kruskal Wallis, respectively. * *P*≤0.05, *** *P*≤0.001.

A small increase in the proportion of control embryos with micronuclei (from 1% to 5%, *P*≤0.05) was observed between collection days 1 and 3, suggesting the formation of new micronuclei by day 3 (Fig. 3*A*). In contrast to this low percentage of control embryos containing micronuclei, the incidence of micronuclei in early cell cleavage embryos fertilized by CPA- exposed males was dramatically increased to 70% (*P*≤0.001, compared to control embryos). CPA treatment did not affect the average number of micronuclei found in micronuclei positive embryos collected on day 1 or 3; when micronuclei were present, there were 2–3 micronuclei per CPA sired embryo as compared to 1–2 micronuclei per control embryo (Fig. 3*B*). Furthermore, micronuclei were distributed randomly into the two daughter cells following the first zygotic division (Fig. 3C, *P*≤0.05). Interestingly, in the CPA sired embryos with micronuclei, both the incidence of micronuclei/embryo and the average number of micronuclei/embryonic cell were significantly increased (*P*≤0.05) in the embryos with a delay in progression (3–4 cells: 79%, 0.9) compared to the normally progressing (5–8 cells: 79%, 0.4) and rapidly dividing (9–16 cells: 46%, 0.2) embryos (Fig. 3*D & E*). Thus, micronuclear formation during the first zygotic division was associated with a developmental delay in early cell cleavage embryos.

### Chromatin compaction in the nuclei and micronuclei of early cleavage stage embryos sired by CPA exposed males

**Figure 4:**
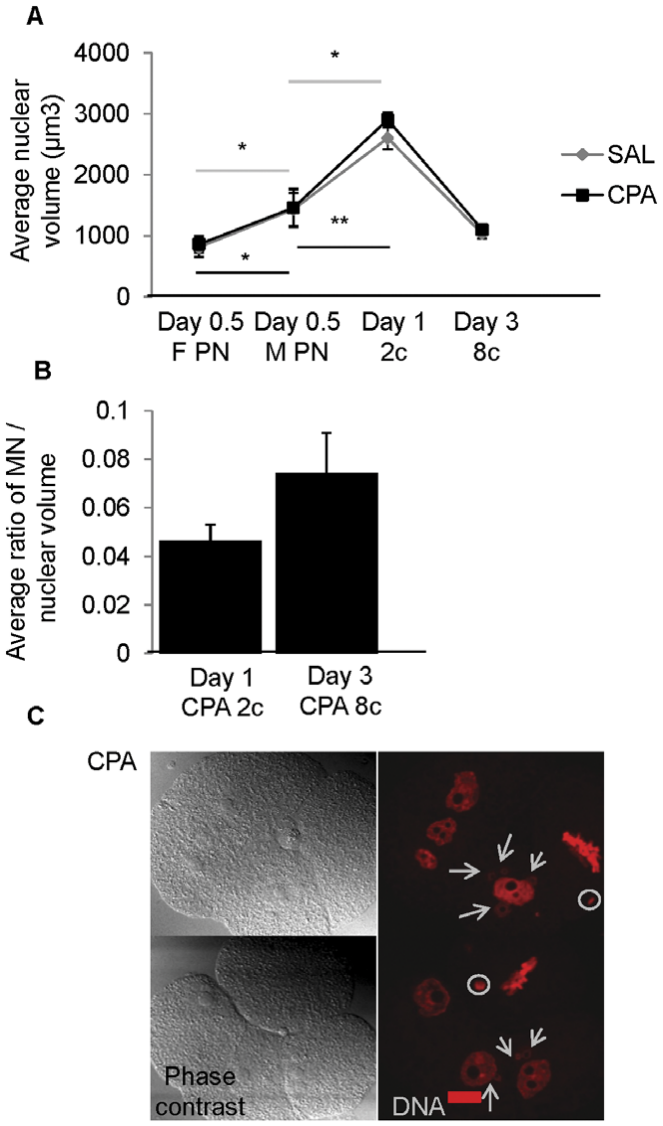
Nuclear and micronuclear (MN) chromatin compaction in early cleavage stage embryos. (A) The nuclear volumes in early cleavage stage control and CPA-sired embryos and the (B) relative ratio of MN to nuclear volumes on collection days 1 and 3 in CPA-sired embryos. Paternal exposure to CPA did not affect the transient nuclear volume changes in early cell cleavage stage embryos and the volume of CPA-induced MN remained comparable to the main nucleus on days 1 and 3 of collection. Line graphs represent the average per replicate and error bars the standard errors of the means; the gray line with diamond symbols designates control and the black line with squares the CPA-sired embryos. (C) Immunofluorescence images of CPA embryos with MN stained with propidium iodide in red to compare the chromatin compaction of MN and nuclei within the same embryonic cell at different mitotic phases. Within the same cell, the MN and nuclear chromatin was condensed during mitosis and decondensed during interphase, suggesting communication. Arrows point towards decondensed MN chromatin while condensed MN chromatin is circled in white. We collected: day 0.5 SAL N = 6 males, 109 embryos, CPA N = 5 males, 84 embryos; day 1 SAL N = 8 males, 115 embryos, CPA N = 5 males, 64 embryos; day 3 SAL N = 8males, 129 embryos, CPA N = 8 males, 120. The Kruskal Wallis test with Bonferroni's correction was performed for statistical analysis * *P*≤0.05, ** *P*≤0.01.

The volumes of the female and male pronuclei (day 0.5) did not differ (Fig. 4*A*); however, the nuclear volumes were doubled in the progression from pronuclear zygote (1440 µm^3^) to 2 cell embryo (2750 µm^3^) (*P*≤0.05). A decrease in nuclear volume was observed as embryos progressed from 2 cell to 8 cell (1070 µm^3^) (*P*≤0.05), returning to the volume in pronuclear zygotes. Paternal exposure to CPA did not affect these transient nuclear volume changes (Fig. 4*A*). The volume ratios as well as the chromatin compaction of the nuclei and micronuclei were assessed only in embryos sired by CPA exposed males since the incidence of micronuclei in control embryos was very low. The volume of micronuclei in CPA sired embryos, as a ratio of the nuclear volume, did not change from day 1 to day 3 (0.05 to 0.07, respectively) (Fig. 4*B*). In addition, the compaction state of the chromatin in micronuclei was similar to that of the main nucleus within each cell, i.e. condensed during mitosis and decondensed during interphase (Fig. 4*C*), suggesting that the micronuclei were responding to signals from the main nucleus or the cytoplasm.

### Micronuclear characterization in early cell cleavage embryos fertilized by CPA exposed males

**Figure 5:**
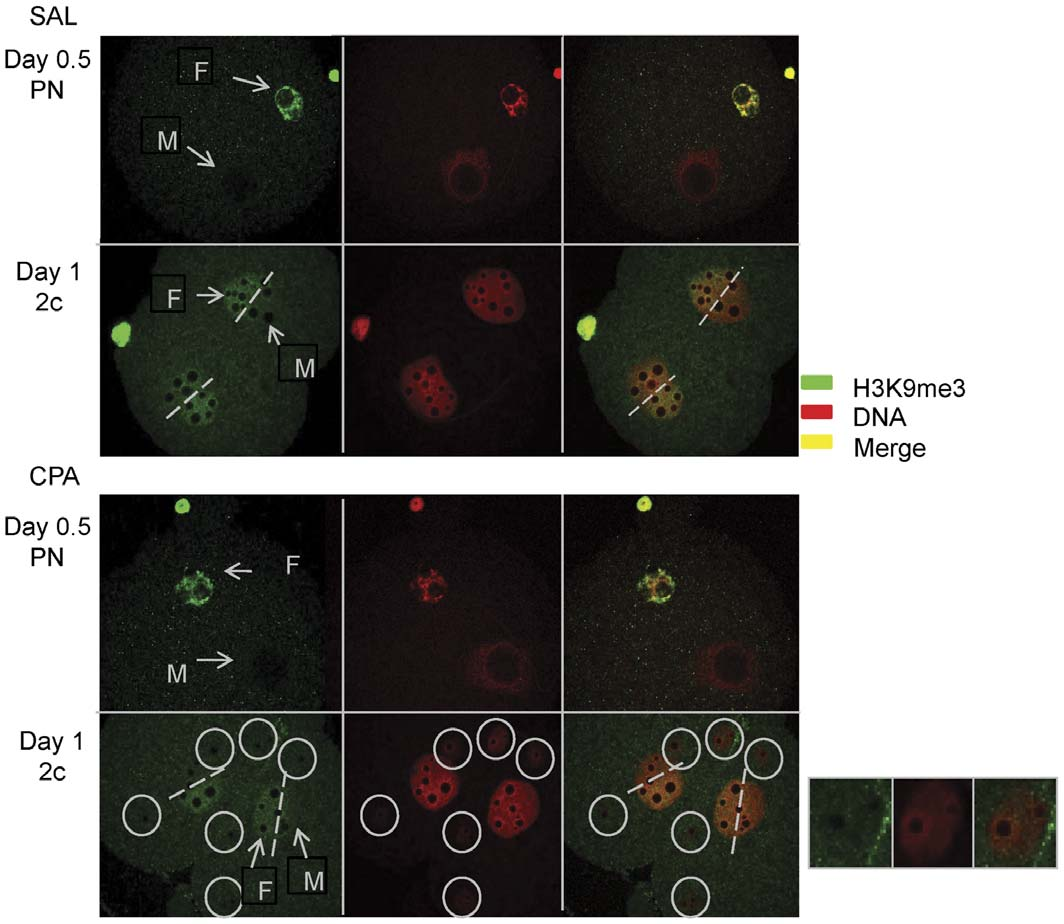
Parental origin of micronuclear chromatin in early cell cleavage embryos fertilized by CPA exposed males. Pronuclear zygotes and 2 cell embryos were stained for the maternal epigenetic chromatin specific mark H3K9me3 (in green), DNA (counterstained with propidium iodide in red) with the merged image in yellow. The top panels are control and the bottom panels are CPA-sired embryos. Only the female pronucleus and polar body stained with H3K9me3 in zygotes; in 2 cell embryos half of each nucleus stained with H3K9me3, representing the chromatin of female origin. Paternal CPA treatment did not affect this parental chromatin mark. The absence of H3K9me3 in MN in CPA-sired embryos indicates that these MN are of paternal chromatin origin in 2 cell embryos. The immunofluorescence images were acquired with a confocal microscope. Arrows point to the Female (F) and Male (M) pronuclei, MN are circled in white, dashed white lines delineate the female (positive signal for H3K9me3) from the male (negative signal for H3K9me3) chromatin in 2 cell embryos; a magnified image of MN is presented on the right side of the CPA-sired 2 cell embryos. We collected SAL and CPA, N = 2–3 males with 15–30 embryos per group per embryonic cell cleavage stage for qualitative analysis.

H3K9me3 is a female specific epigenetic mark. In both control and CPA sired embryos, only the female pronucleus in the zygote and the chromatin of female origin (half of the nucleus) in 2 cell embryos contained this epigenetic mark (Fig. 5); the male pronucleus and the remaining half of the nucleus in 2 cell embryos were negative for H3K9me3 (Fig. 5). The micronuclear chromatin of day 1 embryos fertilized by CPA exposed spermatozoa was always negative for H3K9me3, suggesting that the micronuclei are of paternal origin (Fig. 5, bottom panel).

**Figure 6:**
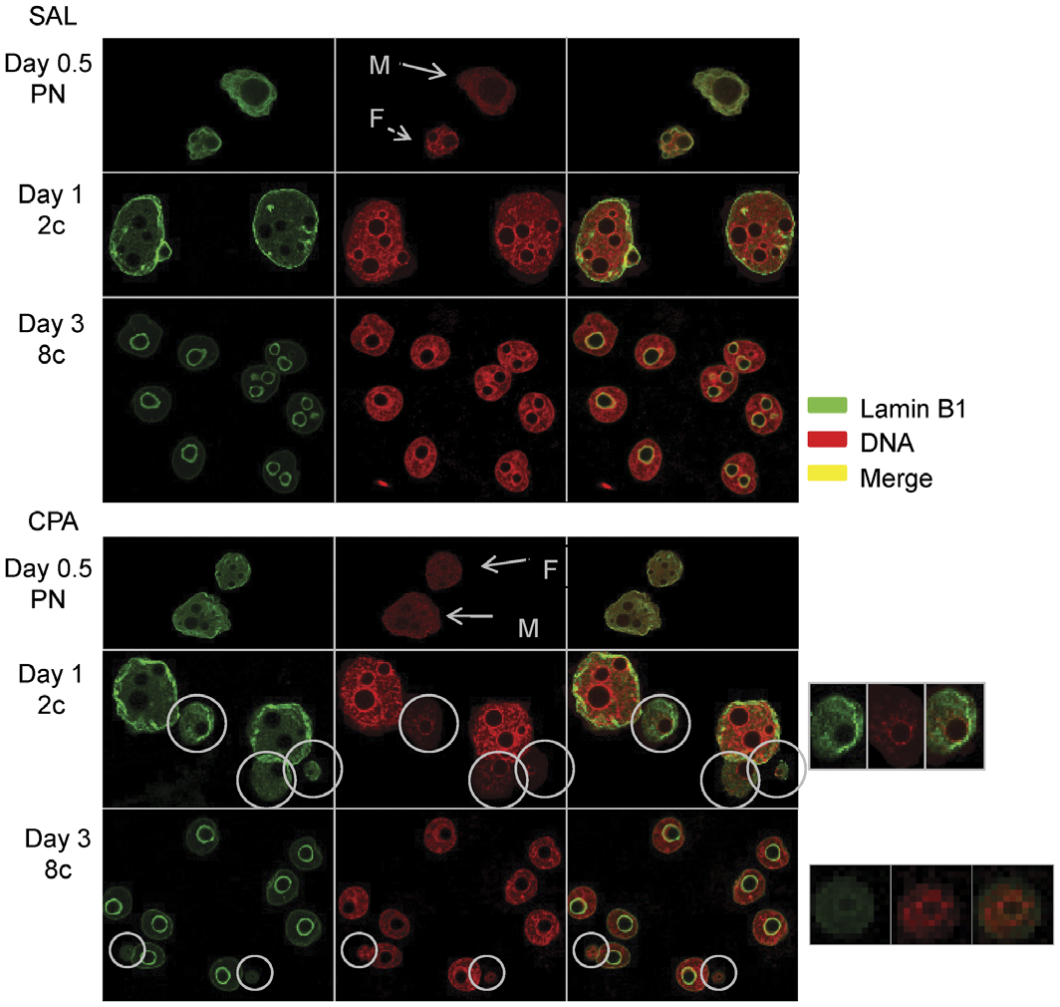
Micronuclear membrane characterization in early cell cleavage embryos fertilized by CPA exposed spermatozoa. Pronuclear zygotes and 2 and 8 cell CPA-sired embryos were collected on days 0.5, 1 and 3 and stained for nuclear membrane lamin B1 (in green), DNA (propidium iodide, in red) with the merged image in yellow. We observed a clear nuclear to peri-nucleolar membrane redistribution of lamin B1 in early 2 and 8 cell embryos, respectively; this was not affected by paternal CPA treatment. MN in CPA-sired embryos showed the formation of incomplete nuclear and peri-nucleolar membranes at both embryonic collection time points. These images were acquired with a confocal microscope. Arrows point to Female (F) and Male (M) pronuclei, MN are circled in white, with a magnified image on the left of each respective embryonic stage. We collected N = 2–3 males, 15–30 embryos per group per embryonic cell cleavage stage for both SAL and CPA for qualitative analysis.

Since lamin proteins are thought to be involved in nuclear stability, chromatin structure and gene expression, we visualized lamin B1 immunoreactivity in control and CPA sired pronuclear, 2 cell and 8 cell embryos. In control pronuclear and 2 cell embryos, a clear peri-nuclear ring was observed (Fig. 6). By the 8 cell stage, lamim B1 immunoreactivity was redistributed, forming an intense peri-nucleolar ring with a much fainter peri-nuclear ring. While the lamin B1 reactivity of the main nucleus in CPA sired embryos was similar to that in the control embryos, the CPA induced micronuclei had much fainter or even absent lamin B1 membrane staining (Fig. 6, bottom panel), suggesting incomplete formation of the micronuclear and their associated micronucleolar membranes.

### DNA replication in the micronuclei of CPA sired embryos

**Figure 7:**
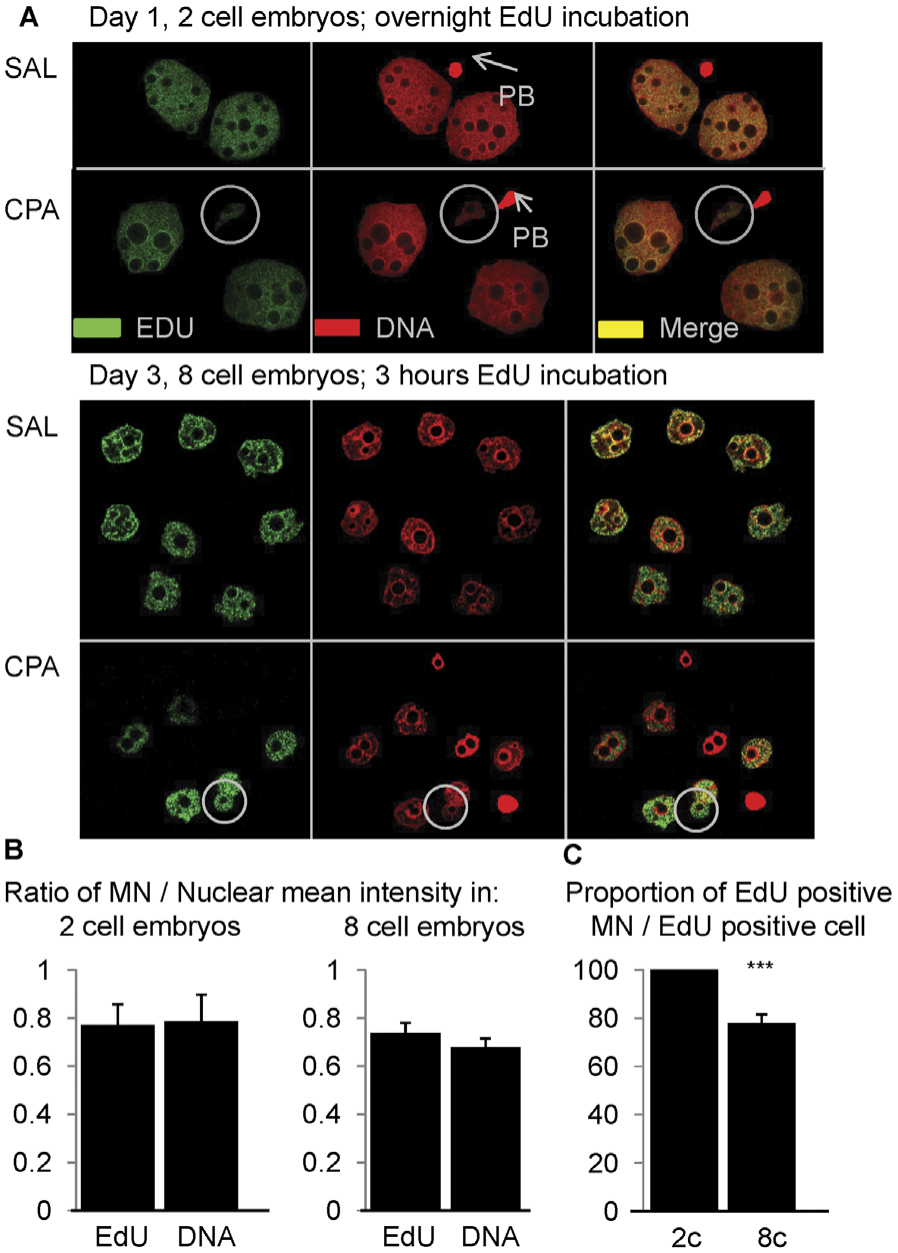
DNA replication in the micronuclei of CPA sired embryos. Immunofluorescence images of embryos stained with EdU Click-it (in green) and DNA (propidium iodide in red) with the merged images in yellow. MN are circled in white, the arrows point to the polar body (PB) (internal negative control for EdU). (A) top panel: overnight *in vitro* incubation during the first zygotic division; bottom panel: 3 h *in vitro* incubation of day 3 embryos with EdU to assess DNA replication in early cell cleavage embryos. Paternal exposure to CPA did not affect on the proportion of cells incorporating EdU in early cell cleavage embryos. (B) Comparison of the MN to nuclear ratios of EdU incorporation relative to DNA content in day 1 and 3 embryos sired by CPA exposed males. We observed that MN synthesized DNA to the same extent as the nuclei in both 2 and 8 cell embryos. (C) Comparison of the proportion of EdU positive MN in EdU positive cells in day 1 and day 3 embryos sired by CPA exposed males. A decrease in the proportion of functional MN incorporating EdU was observed in 8 cell embryos compared to 2 cell embryos. Bar graphs are the average ratio or proportion per replicate; error bars represent the standard errors of the means. We collected on day 1 SAL N = 6 males, 93 embryos CPA N = 5 males, 29 embryos and on day 3 SAL N = 5 males, 55 embryos CPA N = 6 males, 73 embryos. The ratio comparisons were analyzed statistically by Kruskal-Wallis and the proportions of embryos were analyzed statistically by Chi-square, Fisher Exact test and: *** *P*≤0.001.

In vitro incubation of early cell cleavage stage embryos with the EdU click-it kit allowed the visualization of DNA synthesis as a marker of function. EdU was incorporated into all embryonic cells independently of the treatment group, thus the capacity of CPA sired embryos to synthesize DNA as they progressed from the pre-pronuclear zygote to the 2 cell stage did not differ from control (Fig. 7*A*, top panel). Again, at the 8 cell stage, DNA synthesis in control and CPA sired embryos did not differ significantly, although in control embryos 81% of the embryonic cells incorporated EdU, whereas in CPA sired embryos 65% of the cells incorporated EdU (Fig. 7*A*, bottom panel). The observation that all cells did not incorporate EdU within the 3 hr incubation time is not surprising since mitosis is asynchronous between cells.

EdU incorporation in the micronuclei of CPA sired 2 cell embryos (collected on day 1) was the same as that of the main nucleus since the ratio of micronuclear to nuclear EdU reactivity (0.77) was the same as that for the DNA content (0.79) (Fig. 7*B*, left graph). The ratio of EdU reactivity (0.74) to DNA content (0.68) in micronuclei versus the main nucleus in CPA sired 8 cell embryos was also similar (Fig. 7*B*, right graph). However, the proportion of micronuclei in EdU positive cells that were capable of synthesizing DNA decreased significantly, from 100% to 78%, between the 2 and 8 cell stages (Fig. 7*C*, *P*≤0.001).

## Discussion

Lagging, fragmented pieces of DNA at metaphase and telophase during the first zygotic division are a visible mark of DNA damage as a consequence of paternal exposure to CPA; these fragments form micronuclei in 2 cell embryos. Thus, damaged DNA is released from the main nucleus to form micronuclei at the time of the first zygotic division, during chromatin decondensation and removal of the pronuclear membrane. Using an epigenetic mark, we confirmed that these micronuclei represent paternal chromatin. Within an embryo that had micronuclei, it was not unusual to observe some blastomeres with micronuclei and others without; even those blastomeres that did not have micronuclei may have DNA damage.

The presence of micronuclei was associated with a delay in progression through the early cell cleavage stages of development. This delay in progression of the CPA sired embryos was seen as early as the first zygotic division but did not affect the ability of the zygote to reach the 2 cell stage. This initial delay may become greater in later cleavage divisions since a greater proportion of delayed embryos was observed among the CPA treated group on collection day 3 compared to the controls. Interestingly, we have reported that paternal exposure to CPA led to an enhanced rate of sperm DNA decondensation and chromatin remodelling in the pre-pronuclear zygote [22] and an acceleration in progression through the stages of pronuclear zygote development [9]. Thus, damage to the male genome may initially speed up unpackaging of the paternal chromatin after fertilization but subsequently the damage, if it is extensive and is not repaired by embryonic DNA repair processes, has adverse effects on early cell divisions. Previous studies have reported a decrease in cell numbers in preimplantation embryos sired by CPA treated males [8], [27].

A similar relationship between the formation of micronuclei and a delay in embryonic progression was reported following parental exposure to irradiation, other alkylating chemicals and pesticides [28]–[30]. The proportion of embryos with micronuclei is the same from the 2 to 8 cell stage, so all micronuclei are formed during the first zygotic division. Since no new micronuclei are generated in the next two cleavage divisions, the average number of micronuclei per cell decreases as the cell number increases. This suggests that replicated micronuclei segregate randomly into the daughter cells during mitosis, as plasmids do in bacteria. Thus, genetic material is lost to the embryo. This probably contributes to embryo death. Indeed, previous studies established that the CPA treatment regimen used here induced approximately an 80% incidence of peri-implantation loss [6]. The 2 cell embryos that are free of micronuclei may have a reasonable chance to survive while the CPA sired embryos with micronuclei display progressive delays in cell division.

Our data indicate that the extent of DNA damage caused by paternal exposure to CPA, as assessed by the number of micronuclei per embryo, has a direct impact on fate of the embryo. It is likely that CPA sired early cleavage stage embryos have the capacity to undergo a certain number of cell cycles but die around the time of implantation from damage accumulation. It has been suggested that embryonic cell death pathways may be triggered by the activation of DNA damage-sensing checkpoint kinases [31], spindle associated checkpoints [32], an inadequate DNA damage repair response [33], or energy depletion [34] and that at least some of these pathways are p53-dependent [35].

Micronuclear formation and function may differ depending on the type of chemical exposure and the cell type [13]. The micronuclei that are induced in CPA sired embryos during the first zygotic division were very similar to the main nuclei: during interphase, the micronuclear chromatin was decondensed and round with a single nucleolus, while during mitosis the micronuclear chromatin was condensed. In cleavage stage embryos, the micronuclei followed the same transient nuclear volume change exerted on the main nucleus, suggesting that micronuclei are in communication with the main nucleus and cytoplasm within the cell.

The characterization of CPA induced micronuclear chromatin structure was initially done using H3K9me3, a female specific epigenetic mark, in early pronuclear and 2 cell embryos. As anticipated based on previous studies with mouse embryos [36], a clear delineation between the female chromatin and male chromatin was observed in both control and CPA sired 2 cell embryos. H3K9me3, was absent from all of the micronuclei observed in CPA-sired embryos. Thus, the genetic material in the micronuclei is of paternal origin.

Lamin B1 immunoreactivity was localized to the peri-nuclear (pronuclear and 2 cell) and both the peri-nuclear and peri-nucleolar membranes (4 and 8 cell stages) in control and CPA sired embryos. While there was no effect of paternal CPA treatment on the localization of lamin B1, the micronuclei that were induced had incomplete peri-micronuclear and peri-micronucleolar lamin B1 membrane formation. Since lamins play a central role in formation of the nuclear pore complex in cells [37], these data may indicate a disturbance in the communication or exchange processes between the nucleus, micronucleus and cytoplasm within the embryos. Despite this indication of altered structure, the function of micronuclei, as indicated by their ability to incorporate EdU into newly synthesized DNA, was maintained. In addition, we did not observe any correlation between EdU incorporation and development delay in CPA sired embryos.

Micronuclear formation may result from a number of different causes. These include: DNA damage that is either not repaired or misrepaired, hypomethylation of repeat sequences in centromeric and pericentromeric DNA, defects in kinetochore proteins or assembly, or dysfunctional spindle and defective anaphase checkpoint genes [38]. In this context, it is interesting to note that the extensive DNA methylation reprogramming that is crucial for embryogenesis is disrupted in zygotes sired by cyclophosphamide-treated males [9]; the male pronuclei in zygotes fertilized by drug-exposed spermatozoa were dramatically hypomethylated in pronuclear stage 3 embryos. Protein modifications may also play a role since the H3K9me3 methylation mark may be necessary for the connection of microtubules to the kinetochores during mitosis [38]. However, since microtubule and spindle assembly components are of maternal origin, it is most likely that DNA damage, genetic or epigenetic, is responsible for driving the process by which micronuclei are formed in these experiments.

It has been reported that in human embryos generated by assisted reproductive technologies, nearly 60% exhibit chromosomal mosaicism and aneuploidy by the 4 cell stage and over 90% by the blastocyst stage [39], [40]. It is clear that spermatozoa from men who are sub-fertile have an increased likelihood of containing DNA damage [41] as do those from cancer survivors who have received treatment with radiation or DNA damaging chemotherapeutics [3], [42]. Advances in our understanding of the molecular mechanisms involved in the formation of micronuclei and the consequences in terms of changes in the genome, epigenome, transcriptome, and proteome of the early embryo will help to elucidate biomarkers that may indicate the health of the paternal genome.

## References

[1] Huyghe E, Plante P, Thonneau PF (2007). Testicular cancer variations in time and space in Europe.. Eur Urol.

[2] Thomson AB, Campbell AJ, Irvine DC, Anderson RA, Kelnar CJ (2002). Semen quality and spermatozoa DNA integrity in survivors of childhood cancer: a case-control study.. Lancet.

[3] Green DM, Kawashima T, Stovall M, Leisenring W, Sklar CA (2010). Fertility of male survivors of childhood cancer: a report from the Childhood Cancer Survivor Study.. J Clin Oncol.

[4] Stahl O, Boyd HA, Giwercman A, Lindholm M, Jensen A (2011). Risk of birth abnormalities in the offspring of men with a history of cancer: a cohort study using Danish and Swedish national registries.. J Natl Cancer Inst.

[5] Colvin OM (1999). An overview of cyclophosphamide development and clinical applications.. Curr Pharm Des.

[6] Trasler JM, Hales BF, Robaire B (1986). Chronic low dose cyclophosphamide treatment of adult male rats: effect on fertility, pregnancy outcome and progeny.. Biol Reprod.

[7] Kelly SM, Robaire B, Hales BF (1992). Paternal cyclophosphamide treatment causes postimplantation loss via inner cell mass-specific cell death.. Teratology.

[8] Harrouk W, Robaire B, Hales BF (2000). Paternal exposure to cyclophosphamide alters cell-cell contacts and activation of embryonic transcription in the preimplantation rat embryo.. Biol Reprod.

[9] Barton TS, Robaire B, Hales BF (2005). Epigenetic programming in the preimplantation rat embryo is disrupted by chronic paternal cyclophosphamide exposure.. Proc Natl Acad Sci U S A.

[10] Codrington AM, Hales BF, Robaire B (2004). Spermiogenic germ cell phase-specific DNA damage following cyclophosphamide exposure.. J Androl.

[11] Harrouk W, Codrington A, Vinson R, Robaire B, Hales BF (2000). Paternal exposure to cyclophosphamide induces DNA damage and alters the expression of DNA repair genes in the rat preimplantation embryo.. Mutat Res.

[12] Au WW, Giri AK, Ruchirawat M (2010). Challenge assay: A functional biomarker for exposure-induced DNA repair deficiency and for risk of cancer.. Int J Hyg Environ Health.

[13] Terradas M, Martín M, Tusell L, Genescà A (2010). Genetic activities in micronuclei: is the DNA entrapped in micronuclei lost for the cell?. Mutat Res.

[14] Kirsch-Volders M, Plas G, Elhajouji A, Lukamowicz M, Gonzalez L (2011). The in vitro MN assay in 2011: origin and fate, biological significance, protocols, high throughput methodologies and toxicological relevance.. Arch Toxicol.

[15] Sartore RC, Campos PB, Trujillo CA, Ramalho BL, Negraes PD (2011). Retinoic acid-treated pluripotent stem cells undergoing neurogenesis present increased aneuploidy and micronuclei formation.. PLoS One.

[16] Delbes G, Hales BF, Robaire B (2007). Effects of the chemotherapy cocktail used to treat testicular cancer on sperm chromatin integrity.. J Androl.

[17] O'Flaherty C, Vaisheva F, Hales BF, Chan P, Robaire B (2008). Characterization of sperm chromatin quality in testicular cancer and Hodgkin's lymphoma patients prior to chemotherapy.. Hum Reprod.

[18] Trasler JM, Hales BF, Robaire B (1985). Paternal cyclophosphamide treatment of rats causes fetal loss and malformations without affecting male fertility.. Nature.

[19] Marchetti F, Bishop J, Lowe X, Wyrobek AJ (2009). Chromosomal mosaicism in mouse two-cell embryos after paternal exposure to acrylamide.. Toxicol Sci.

[20] Yamagata K, Suetsugu R, Wakayama T (2009). Assessment of chromosomal integrity using a novel live-cell imaging technique in mouse embryos produced by intracytoplasmic sperm injection.. Hum Reprod.

[21] Nygren KG, Sullivan E, Zegers-Hochschild F, Mansour R, Ishihara O (2011). International Committee for Monitoring Assisted Reproductive Technology (ICMART) world report: assisted reproductive technology 2003.. Fertil Steril.

[22] Grenier L, Robaire B, Hales BF (2010). Paternal exposure to cyclophosphamide affects the progression of sperm chromatin decondensation and activates a DNA damage response in the prepronuclear rat zygote.. Biol Reprod.

[23] Oh SH, Miyoshi K, Funahashi H (1998). Rat oocytes fertilized in modified rat 1-cell embryo culture medium containing a high sodium chloride concentration and bovine serum albumin maintain developmental ability to the blastocyst stage.. Biol Reprod.

[24] van der Heijden GW, Dieker JW, Derijck AAHA, Muller S, Berden JHM (2005). Asymmetry in Histone H3 variants and lysine methylation between paternal and maternal chromatin of the early mouse zygote.. Mech Dev.

[25] Hutchison CJ (2002). Lamins: building blocks or regulators of gene expression?. Nat Rev Mol Cell Biol.

[26] Broers JL, Machiels BM, Kuijpers HJ, Smedts F, van den Kieboom R (1997). A- and B-type lamins are differentially expressed in normal human tissues.. Histochem Cell Biol.

[27] Austin SM, Robaire B, Hales BF, Kelly SM (1994). Paternal cyclophosphamide exposure causes decreased cell proliferation in cleavage-stage embryos.. Biol Reprod.

[28] Mozdarani H, Nazari E (2009). Cytogenetic damage in preimplantation mouse embryos generated after paternal and parental gamma-irradiation and the influence of vitamin C.. Reproduction.

[29] Tian Y, Yamauchi T (2003). Micronucleus formation in 3-day mouse embryos associated with maternal exposure to chlorpyrifos during the early preimplantation period.. Reprod Toxicol.

[30] Titenko-Holland N, Ahlborn T, Lowe X, Shang N, Smith MT (1998). Micronuclei and developmental abnormalities in 4-day mouse embryos after paternal treatment with acrylamide.. Environ Mol Mutagen.

[31] Mu XF, Jin XL, Farnham M, Li Y, O'Neill C (2011). DNA Damage-Sensing Kinases Mediate the Mouse Two-Cell Embryo's Response to Genotoxic Stress.. Biol Reprod.

[32] Wei Y, Multi S, Yang CR, Ma J, Zhang QH (2011). Spindle Assembly Checkpoint Regulates Mitotic Cell Cycle Progression during Preimplantation Embryo Development.. PLoS ONE.

[33] Yukawa M, Oda S, Mitani H, Nagata M, Aoki F (2007). Deficiency in the response to DNA double-strand breaks in mouse early preimplantation embryos.. Biochem Biophys Res Commun.

[34] Wakefield SL, Lane M, Mitchell M (2011). Impaired mitochondrial function in the preimplantation embryo perturbs fetal and placental development in the mouse.. Biol Reprod.

[35] Toyoshima M (2009). Analysis of p53 dependent damage response in sperm-irradiated mouse embryos.. J Radiat Res (Tokyo).

[36] Santenard A, Ziegler-Birling C, Koch M, Tora L, Bannister AJ (2010). Heterochromatin formation in the mouse embryo requires critical residues of the histone variant H3.3.. Nat Cell Biol.

[37] Fiserova J, Goldberg MW (2010). Relationships at the nuclear envelope: lamins and nuclear pore complexes in animals and plants.. Biochem Soc Trans.

[38] Fenech M, Kirsch-Volders M, Natarajan AT, Surralles J, Crott JW (2011). Molecular mechanisms of micronucleus, nucleoplasmic bridge and nuclear bud formation in mammalian and human cells.. Mutagenesis.

[39] Sandalinas M, Sadowy S, Alikani M, Calderon G, Cohen J (2001). Developmental ability of chromosomally abnormal human embryos to develop to the blastocyst stage.. Hum Reprod.

[40] Bielanska M, Tan SL, Ao A (2002). Chromosomal mosaicism throughout human preimplantation development in vitro: incidence, type, and relevance to embryo outcome.. Hum Reprod.

[41] Zini A (2011). Are sperm chromatin and DNA defects relevant in the clinic?. Syst Biol Reprod Med.

[42] O'Flaherty C, Hales BF, Chan P, Robaire B (2010). Impact of chemotherapeutics and advanced testicular cancer or Hodgkin lymphoma on sperm deoxyribonucleic acid integrity.. Fertil Steril.

